# Laparoscopic versus open peritoneal dialysis catheter insertion, the LOCI-trial: a study protocol

**DOI:** 10.1186/1471-2482-11-35

**Published:** 2011-12-20

**Authors:** Sander M Hagen, Arjan M van Alphen, Jan NM IJzermans, Frank JMF Dor

**Affiliations:** 1 Department of Surgery, Erasmus MC, University Medical Center, Rotterdam, The Netherlands. PO BOX 2040, 3000 CA, Rotterdam, The Netherlands; 2 Department of Nephrology, Erasmus MC, University Medical Center, Rotterdam, The Netherlands

## Abstract

**Background:**

Peritoneal dialysis (PD) is an effective treatment for end-stage renal disease. It allows patients more freedom to perform daily activities compared to haemodialysis. Key to successful PD is the presence of a well-functioning dialysis catheter. Several complications, such as in- and outflow obstruction, peritonitis, exit-site infections, leakage and migration, can lead to catheter removal and loss of peritoneal access. Currently, different surgical techniques are in practice for PD-catheter placement. The type of insertion technique used may greatly influence the occurrence of complications. In the literature, up to 35% catheter failure has been described when using the open technique and only 13% for the laparoscopic technique. However, a well-designed randomized controlled trial is lacking.

**Methods/Design:**

The LOCI-trial is a multi-center randomized controlled, single-blind trial (pilot). The study compares the laparoscopic with the open technique for PD catheter insertion. The primary objective is to determine the optimum placement technique in order to minimize the incidence of catheter malfunction at 6 weeks postoperatively. Secondary objectives are to determine the best approach to optimize catheter function and to study the quality of life at 6 months postoperatively comparing the two operative techniques.

**Discussion:**

This study will generate evidence on any benefits of laparoscopic versus open PD catheter insertion.

**Trial registration:**

Dutch Trial Register NTR2878

## Background

Approximately 15.000 patients in the Netherlands are diagnosed with end-stage renal disease (ESRD) and are dependent on renal replacement therapy (peritoneal dialysis, haemodialysis or transplantation). Almost 6.300 patients are on dialysis. One fifth is on peritoneal dialysis (PD). PD has several advantages over haemodialysis (HD): it allows patients greater freedom to perform daily activities, it requires fewer dietary restriction, and mortality is lower during the first two years of treatment [1]. The costs may also be of great importance. PD costs up to $43K dollars less than HD per person per year, therefore well-functioning PD may have major economic consequences [2,3]. However, we have noticed that PD catheter insertion has a high rate of technical failure using the standard open technique. Case-series report up to 35% catheter failure with the open technique [4-9]. Catheter malfunction is most commonly caused by complications, such as malpositioning of the catheter tip, in- and outflow obstruction, peritonitis, exit-site infections and leakage. These complications often lead to re-operation and even loss of PD as dialysis modality. For a small but significant number of patients this results in severe morbidity and even mortality. For many other surgical procedures, laparoscopy has proven to be superior to the open techniques, by reducing morbidity, length of hospital stay, postoperative pain and improved convalescence [10-13]. An advantage of laparoscopic PD catheter insertion, compared to the conventional open technique, is the ability to insert the catheter under direct vision. Direct visual feedback during placement, leads to better positioning at the end of the operation [14,15].

In current literature, comparative trials reported no significant difference in the risk of catheter removal, replacement or technical failure between both techniques [9,16-19], however there are no well-designed randomized controlled trials comparing laparoscopic PD catheter insertion to the traditional open technique.

## Methods/Design

### Study objectives

Primary Objective: Does the use of the laparoscopic insertion technique lower the incidence of malfunctioning PD catheters at 6 weeks postoperatively?

Secondary Objectives: Does the use of the laparoscopic insertion technique improve catheter longevity and reduce the rate of surgical complications, mortality, leakage, catheter migration, re-admissions, exit-site infections, peritonitis and duration of hospital stay? Does the use of the laparoscopic insertion technique reduce postoperative pain, the use of postoperative pain medication and increase the quality of life? Does the use of the laparoscopic insertion technique lower the incidence of malfunctioning PD catheters at 6 months postoperatively?

### Study design

The LOCI-trial is a multicenter prospective single blinded, randomized controlled trial. The LOCI-trial has a pilot nature, as the study will generate data that should enable us to design a large multi-center randomised controlled trial comparing the laparoscopic PD insertion technique with the open technique.

The design of this protocol is in accordance with the CONSORT guideline [20]. We have stratified per center, PD in the past and previous episodes of peritonitis. The study has started on May 24^th ^2011 and the duration will be approximately 1.5 years. This study compares laparoscopic and open PD catheter insertion. In total 100 patients will be included in the study. Approval of the Medical Ethical Committee Erasmus MC, Rotterdam, The Netherlands, was obtained.

Randomisation will take place after endotracheal intubation, by means of a web-based computer programme with supervision of the study coordinator. This computer programme will generate the randomisation sequence. The patient will be blinded for the operation technique in the postoperative period. The wounds will be covered with a standard pattern of bandages [21]. All patients will fill out questionnaires (the Short-Form 36 (SF-36), EuroQol (EQ-5D) and a Visual Analogue Scale (VAS) for pain) until 6 months postoperatively. (Table 1)

**Table 1:** Time schedule for filling out the questionnaires

Time of evaluation	VAS	EuroQol	SF-36
Preoperative	X	X	X
Postoperative			
Day 0	X		
Day 1	X		
Day 2	X		
Day 3	X	X	
Week 1	X	X	
Week 2	X	X	
Week 4		X	X
Week 6			X
Week 8			X
Week 12		X	X
Week 26		X	X

### Patient selection

All, Dutch speaking, patients with an indication for PD can be included in this trial. Exclusion criteria are: BMI >35 kg/m^2^, age <18 years and patients who are not able to withstand a laparoscopic procedure. Informed consent is mandatory.

Patients will be informed about this study in the outpatient clinic. Patients can contact the research fellow, a surgeon or an independent physician for further information. If patients will not sign the informed consent form, the PD catheter will be inserted via the open technique (considered standard of care in our center).

### Hypothesis

The laparoscopic PD catheter insertion technique will lead to a lower incidence of malfunctioning catheters at 6 weeks postoperatively.

### Study questions

Primary question: Does the use of the laparoscopic insertion technique lower the incidence of malfunctioning PD catheters at 6 weeks postoperatively?

Secondary questions: Does the use of the laparoscopic insertion technique improve catheter longevity and reduce the rate of surgical complications, mortality, leakage, catheter migration, re-admissions, exit-site infections, peritonitis and duration of hospital stay? Does the use of the laparoscopic insertion technique reduce postoperative pain, the use of postoperative pain medication and increase the quality of life? Does the use of the laparoscopic insertion technique lower the incidence of malfunctioning PD catheters at 6 months postoperatively?

### Sample size calculation

At present, we do not have sufficient data to perform a power analysis. Therefore, this pilot study will include two groups of 50 patients. We anticipate that these 100 patients will be sufficient to indicate a difference in technical failure rate at 6 weeks. We do not expect to find significant differences in secondary end points in this relatively small number of patients. In order to be able to include the number of required patients, this study will take place in multiple hospitals. Based on the outcome of this pilot study, we will design a larger multicenter randomized controlled trial, including a large number of patients based on a power calculation.

### Surgical interventions

Laparoscopic technique: pre-operatively, the surgeon or peritoneal dialysis nurse will have marked the exit place of the catheter together with the patient (left or right), well above the belt.

The patient will be operated under general anaesthesia. Antibiotic prophylaxis (Vancomycin 1000 mg IV) will be administered on the ward approximately 1 hour before incision. Chloorhexidine desinfection will routinely be applied. The patient will be covered with sterile drapes. The patient is put in Trendelenburg position. A small subumbilical incision will be made, the fascia will be opened. A 10-12 mm balloon trocar will be inserted and pneumoperitoneum will be created by gas insufflation. Using a 30 degrees optique, the peritoneal cavity will be inspected. Adhesions will be scored and adhaesiolysis will be performed where necessary to place the catheter. A double-cuffed Swan Neck Tenckhoff dialysis catheter will be placed on the abdomen of the patient, to determine the best entry place and exit point. A small incision is made at the entry point. With an 8 mm trocar, a subcutaneous tunnel is created. The trocar is then introduced in the peritoneal cavity. The catheter will now be introduced with a stylet, without twisting the catheter around the stylet. If necessary, an additional 5 mm trocar is inserted to enable securing of the PD catheter in correct position. The catheter tip will be placed in the pouch of Douglas. The stylet will be retracted and the 8 mm trocar will be removed. The distal cuff of the catheter remains just outside the peritoneum. The peritoneal cavity will be desufflated. The inflow and outflow will be tested with at least 500 cc of saline with the patient in neutral position. This should be very easy. Then the balloon trocar will be removed. The sub umbilical fascia will be closed with Vycril-UR6 and the closure of the skin with Monocryl. After 14 days, PD training can be commenced. Conversion to an open procedure will be performed for indications that are common in laparoscopic procedures.

Open technique: The pre-operative measures are the same as in the experimental intervention. A 4-5 cm pararectal incision will be made, the anterior rectus fascia will be opened, the muscles will be split and the dorsal rectus fascia will be opened. The surgeon ensures that the surrounding peritoneum is free of adhesions with his fingers. Preferably, the os pubis is felt. The catheter will now be introduced as described above and the tip will be placed in the pouch of Douglas. Testing of in- and outflow will be as described above. The peritoneum and fascia will be closed with a purse string suture using PDS 3-0. The proximal end of the catheter will be connected to a Redon needle, and a natural exit point is determined, ensuring that the proximal cuff is far enough from the exit point. Further as above. As with the laparoscopic technique, PD training will be started after 14 days.

### Outcome measures

#### Primary outcome

The percentage of functioning CAPD catheters at 6 weeks postoperatively. This is defined by not having an indication for catheter removal or revision for:

• An in- and/or outflow restriction. Outflow restriction is defined as an outflow time longer than 30 minutes, and urokinase treatment has failed.

• Refractory peritonitis, relapse peritonitis or fungal peritonitis, as described in the national guidelines defined by the Dutch Federation of Nephrology.

• Severe abdominal pain (VAS > 8) for 4 weeks.

• Pericanullar or subcutaneous leakage of peritoneal fluid not improving spontaneously within 5 weeks after catheter insertion.

• Tunnel- or exit-site infection not responding to treatment in 2 weeks (refractory infection).

#### Secondary outcomes

Catheter longevity, the rate of surgical complications, mortality, leakage, catheter migration, re-admissions, infections, and duration of hospital stay. The quality of life and pain score. The use of postoperative pain medication. Percentage of functioning PD catheters at 6 months postoperatively.

### Treatment of participating PD patients

All patients will be treated according to the intention to treat principle. Treatment and outpatient clinic visits will be in accordance with the current standard protocol. Patients will be asked to fill out different standardised questionnaires to evaluate pain and nausea (VAS-score), and quality of life (SF-36 and EQ-5D). (Table 1)

### Statistical analysis

#### Descriptive statistics

Categorical variables will be presented as numbers (percentages). Continuous variables will be presented as medians (ranges). Categorical variables will be compared with the Chi-square test. Continuous variables will be compared with the Mann-Whitney-U test. All analyses will be conducted using SPSS (version 17.0, SPSS Inc., Chicago, USA). A P-value <0.05 (two-sided) will be considered statistically significant.

#### Univariate analysis

Categorical variables will be compared with the Chi-square test. Continuous variables will be compared with the Mann-Whitney-U test or the two sample unpaired t-test.

#### Multivariate analysis

Logistic regression will be applied to determine the independent effects of age, gender, ASA classification, body mass index, reason ESRD, time of surgery, medical history, previous PD catheter insertion and previous abdominal surgery on catheter survival.

### Data collection and access to personal data

The operative data will be filled in immediately after the operation by the operating surgeon via an online case record form. The follow-up data will be collected by the study coordinator, using the same system. The case record forms are only accessible by logging in to a specially designed website (http://www.locitrial.nl). All personal data is coded into numbers (1 to 100). Data will be verified at six months postoperatively by comparing the patient records with the completed case record forms manually. The coordinating investigator and the principal investigator are the only persons who have access to the coding system. According to hospital guidelines, all data are imported into a secured database on a server of our institution and are managed by the coordinating investigator. At the end of the trial all data is analysed in collaboration with the trial statistician.

## Discussion

In the literature, case-series suggest that the laparoscopic insertion technique is to be preferred over the open technique. A higher incidence of exit-site infections in the open group (6.3-41% [22-25]) versus the laparoscopic group (2.5-18% [26,27]) is reported, as well as a higher incidence of catheter migration in the open group (7.6-17.1% [22,25,28] versus 1.3-5.4% [26,27,29]. No differences have been described between open and laparoscopic PD catheter insertion regarding the incidence of peritonitis, 2.9-31% [22-25] and 2.5-31% [26,27,30], respectively. Important outcome measures such as cost-effectiveness and quality of life have hitherto not been investigated. To evaluate the true value of laparoscopy in PD-catheter insertion, a well designed randomized controlled trial, such as the LOCI trial, is warranted.

To improve the reporting of this randomized controlled trial, the methods of this protocol are in adherence with the latest CONSORT statement (items 3-12 of the CONSORT checklist [20]). This study is designed as a randomized controlled trial (item 3) and the eligibility criteria for participants are specifically mentioned in the methods section (item 4). The surgical techniques are described in detail, so replication will be possible (item 5). Primary and secondary outcome measures are pre-defined (item 6). This is a pilot-trial and therefore a sample-size calculation is not required at this point (item 7). The methods of patient inclusion, the methods of randomization and the mechanism to conceal the sequence until interventions will be assigned, is described in detail (item 8-10). This study is single-blinded and the methods to achieve this are described in detail (item 11). The statistical methods which will be used for the analysis of the results are present in this protocol (item 12).

## Competing interests

The authors declare that they have no competing interests.

## Authors' contributions

SMH and FJMFD drafted the manuscript and designed the study. AMA and JNMIJ made substantial contributions to the study design and co-authored the writing of the manuscript and revised it critically for important intellectual content. All authors have read and approved the final manuscript.

This project is funded by an efficiency grant of the Erasmus MC (project number: 102542). The funding body had no role in designing or writing this manuscript.

## Pre-publication history

The pre-publication history for this paper can be accessed here:

http://www.biomedcentral.com/1471-2482/11/35/prepub
